# Supplementary material for: Content-rich biological network constructed by mining PubMed abstracts
Source: BMC Bioinformatics. 2004 Oct 8;5:147. doi: 10.1186/1471-2105-5-147 (PMC528731; doi:10.1186/1471-2105-5-147)
Supplement: Additional File 2 — The original results of the above study (non-essential files are deleted to keep the file size under the limit set by BMC bioinformatics). [file 1471-2105-5-147-S2.bz2 › chilibotAdditionalFile2/dip20/34ID7566124E118/html/CASP3_GZMB.html]

 


 **CASP3** and **GZMB** 
  
Found 75 abstracts in PubMed, retrieved 20.  
 

 What does Google say? 
 PDF only 
| .edu only 

---

**Interactive relationship** (e.g. stimulation, inhibition, etc)

**Inhibitory relationship**- Thus granzyme B  [ **GZMB** ]  mediates direct cleavage of caspase 3  [ **CASP3** ]  and also activates mitochondrial disruption, resulting in the release of proapoptotic proteins that suppress caspase inhibition.  Ref: 12648453 Immunity, 2003
**Neutral relationship**- Once in the cytosol, granzyme B  [ **GZMB** ]  targets caspase 3  [ **CASP3** ]  directly or indirectly through the mitochondria, initiating the caspase cascade to DNA fragmentation and apoptosis.  Ref: 12752668 Immunol Rev, 2003
- Caspase 3  [ **CASP3** ]  activation occurred in CC531s cells after perforin granzyme B  [ **GZMB** ]  treatment, but not after the addition of recombinant FasL.  Ref: 11459173 Cancer Immunol Immunother, 2001
- Recent work shows that granzyme B  [ **GZMB** ]  mediated release of apoptotic factors from the mitochondria is essential for the full activation of caspase 3  [ **CASP3** ] .  Ref: 12752668 Immunol Rev, 2003

**Non-interactive relationship** (e.g. studied together, co-existance, homology, etc.)

- In vitro translated AHNAK fragment could be cleaved by granzyme B  [ **GZMB** ]  and caspase 3  [ **CASP3** ] .  Ref: 11866458 Biochem Biophys Res Commun, 2002
- Actual execution of apoptosis depends on proper functioning of effector caspases, particularly caspase 3  [ **CASP3** ] , and on the expression levels of apoptosis regulating proteins, including Bcl 2 and the recently identified granzyme B  [ **GZMB** ]  specific protease inhibitor 9 PI9.  Ref: 12036886 Blood, 2002
- The DS brain was examined using immunocytochemistry and antibodies against the active fragment of caspase 8 AC, 8 and to caspase 3  [ **CASP3** ]  cleavage products of fodrin CCP  [ **GZMB** ] , a neuronal cytoskeleton protein.  Ref: 12213643 Neurosci Lett, 2002
- The aims of this study were to determine whether CC cells are also resistant to perforin granzyme B  [ **GZMB** ]  and whether the FasL resistance lies upstream of caspase 3  [ **CASP3** ]  activation.  Ref: 11459173 Cancer Immunol Immunother, 2001
- The apoptosis associated marker Fas, FasL, caspase 3  [ **CASP3** ]  and granzyme B  [ **GZMB** ]  were expressed by morphologically neoplastic cells in CTCL and by large atypical cells in LyP, with no significant differences.  Ref: 11899146 Br J Dermatol, 2001
- Immunohistochemical demonstration of the expression of th following markers was performed on formalin fixed skin sections Fas, Fas ligand bcl 2, granzyme B  [ **GZMB** ] , the tumour suppressor protein PTEN and the effector caspase, caspase 3  [ **CASP3** ] .  Ref: 11899146 Br J Dermatol, 2001
- Anti beta fodrin Abs in patients sera as well as mouse polyclonal sera raised against the amino terminal beta fodrin fragment did not react with intact beta fodrin, but recognized the 65 kDa amino terminal fragment generated through cleavage by caspase 3  [ **CASP3** ]  or granzyme B  [ **GZMB** ] .  Ref: 11673564 J Immunol, 2001
- Since both the FasL and perforin granzyme B  [ **GZMB** ]  pathways converge at caspase 3  [ **CASP3** ] , we measured caspase 3  [ **CASP3** ]  activity to learn whether the FasL resistance was due to failure to activate this crucial executioner.  Ref: 11459173 Cancer Immunol Immunother, 2001
- Together, these results suggest that CC cells are sensitive to perforin granzyme B  [ **GZMB** ]  induced apoptosis by activating caspase 3  [ **CASP3** ]  and FasL resistance lies upstream of this executioner caspase.  Ref: 11459173 Cancer Immunol Immunother, 2001
- Treatment of HL 60 cells with lactacystin, a selective inhibitor of the proteasome, exponentially increased caspase 3  [ **CASP3** ]  like hydrolytic activity and induced apoptosis but had little or no effect on the activity of upstream caspase 8, caspase 9, or granzyme B  [ **GZMB** ] .  Ref: 12869638 Mol Pharmacol, 2003
- Furthermore, we showed that caspase 3  [ **CASP3** ]  activity is involved in the execution of perforin granzyme B  [ **GZMB** ]  induced apoptosis in CC531 s cells, since the cell permeable caspase 3  [ **CASP3** ]  inhibitor Z DEVD FMK abrogated DNA fragmentation.  Ref: 11459173 Cancer Immunol Immunother, 2001
